# Risk Factors for SARS-CoV-2 Infection and Illness in Cats and Dogs^1^

**DOI:** 10.3201/eid2806.220423

**Published:** 2022-06

**Authors:** Dorothee Bienzle, Joyce Rousseau, David Marom, Jennifer MacNicol, Linda Jacobson, Stephanie Sparling, Natalie Prystajecky, Erin Fraser, J. Scott Weese

**Affiliations:** Ontario Veterinary College, Guelph, Ontario, Canada (D. Bienzle, J. Rousseau, D. Marom, J. MacNicol, J.S. Weese);; Toronto Humane Society, Toronto, Ontario, Canada (L. Jacobson); Toronto Animal Services, Toronto (S. Sparling);; BC Centre for Disease Control Public Health Laboratory, Vancouver, British Columbia, Canada (N. Prystajecky);; University of British Columbia, Vancouver (N. Prystajecky);; Communicable Disease and Immunization Service, BC Centre for Disease Control, Vancouver (E. Fraser);; University of British Columbia School of Population and Public Health, Vancouver (E. Fraser)

**Keywords:** COVID-19, coronavirus disease, SARS-CoV-2, ELISA, severe acute respiratory syndrome coronavirus 2, viruses, respiratory infections, human-animal bond, pets, seropositivity, respiratory, zoonoses, Canada, *Suggested citation for this article*: Bienzle D, Rousseau J, Marom D, MacNicol J, Jacobson L, Sparling S, et al. Risk factors for SARS-CoV-2 infection and illness in cats and dogs. Emerg Infect Dis. 2022 June [*date cited*]. https://doi.org/10.3201/eid2806.220423

## Abstract

We tested swab specimens from pets in households in Ontario, Canada, with human COVID-19 cases by quantitative PCR for SARS-CoV-2 and surveyed pet owners for risk factors associated with infection and seropositivity. We tested serum samples for spike protein IgG and IgM in household pets and also in animals from shelters and low-cost neuter clinics. Among household pets, 2% (1/49) of swab specimens from dogs and 7.7% (5/65) from cats were PCR positive, but 41% of dog serum samples and 52% of cat serum samples were positive for SARS-CoV-2 IgG or IgM. The likelihood of SARS-CoV-2 seropositivity in pet samples was higher for cats but not dogs that slept on owners’ beds and for dogs and cats that contracted a new illness. Seropositivity in neuter-clinic samples was 16% (35/221); in shelter samples, 9.3% (7/75). Our findings indicate a high likelihood for pets in households of humans with COVID-19 to seroconvert and become ill.

SARS-CoV-2 originated in horseshoe bats and probably reached humans through an unidentified intermediary host (*1*). The virus is aerosolized and highly transmissible among humans; new variants have arisen and spread in successive waves across the world since late 2019. Since a report of SARS-CoV-2 infection in a dog in March 2020 (*2*), an ever-increasing range of species has been shown to be susceptible to infection, including household cats, dogs, ferrets, and hamsters (*3*–*10*).

Companion animals have closest contact with humans, creating ample opportunity for exposure. Experimental infections have suggested that most companion animals are infected only transiently, as indicated by PCR positivity or virus isolation (*11*,*12*). Conversely, detection of antibodies by ELISA or neutralizing antibody assay suggests infection rates of 0.2%–43.9% related to factors such as the likelihood and frequency of interaction with infected humans (*13*–*16*). Infections in animals are typically subclinical or associated with transient respiratory or gastrointestinal disease (*17*,*18*). In rare cases, death has been attributed to SARS-CoV-2 infection; however, defining the contribution of SARS-CoV-2 to death in animals with underlying conditions such as cancer, bacterial pneumonia, or obesity is challenging. On the other hand, minks are highly susceptible to infection and pneumonia, and mortality rates of 35%–55% caused by SARS-CoV-2 infection were reported from outbreaks among farmed mink in Utah (*19*). Captive minks also contracted viruses with a unique amino acid substitution in the spike (S) protein that were subsequently retransmitted to humans and to community cats and dogs, around mink farms in the Netherlands (*5*,*20*). Similarly, infected pet Syrian hamsters may also retransmit SARS-CoV-2 to humans (*21*). More than 30% of free-ranging white-tailed deer tested in Ohio were SARS-CoV-2 positive by PCR, and a similarly high proportion of white-tailed deer in Texas and other North America locations had neutralizing antibodies (*22*,*23*). Experimentally, white-tailed deer transmitted SARS-CoV-2 to other deer vertically and horizontally by direct contact (*24*). It has not yet been determined if infected deer experience illness or have increased illness and death rates or if transmission is sustained among wild deer populations. However, such high prevalence suggests SARS-CoV-2 may become endemic in some deer populations in North America.

SARS-CoV-2 is transmitted predominantly via aerosols, aided by proximity of infected and susceptible hosts, the degree of host susceptibility, and the concentration of infectious virions in air. Although most infections in animals originate from humans, neither risk factors for zoonotic transmission from humans to pets nor the frequency and nature of clinical illness in pets are well defined. We report the frequency of SARS-CoV-2 seropositivity in cohorts of pets from households, low-cost neuter clinics, and animal shelters in Ontario, Canada, and analyze household risk factors associated with seropositivity. The University of Guelph (Ontario, Canada) approved the studies by Animal Utilization Protocol 4411 and Research Ethics Board Protocol 20-04-002.

## Methods

### Swab Samples

Pet owners who had a diagnosis of SARS-CoV-2 infection or symptoms compatible with COVID-19 in the previous 3 weeks were invited to have their pet swabbed by study veterinarians during April 24, 2020–August 31, 2021. Dogs, cats, and ferrets of any age and clinical status were eligible for testing; the only exclusion criterion was medical or behavioral issues that precluded safe sampling. We obtained swab samples from the distal nares, oropharynx, and rectum, whenever possible. We placed swabs in inactivating media (PrimeStore; Longhorn Vaccines and Diagnostics, https://lhnvd.com) for a minimum of 12 hours, extracted RNA using Galvens Viral RNA Extraction (Montreal Biotech, https://www.montreal-biotech.com), and eluted into water.

We performed quantitative reverse transcription PCR to amplify SARS-CoV-2 cDNA with primers and probe in the viral N1 gene (Appendix 1). We submitted samples with positive results for amplification of segments of the envelope (E) and RNA-dependent RNA polymerase (RdRp) genes and whole-genome sequencing in additional laboratories.

### Serum Samples

During June 8, 2020–November 30, 2021, we invited owners of pets who received a diagnosis of SARS-CoV-2 infection 2 weeks–3 months previously to have a blood sample of their pet analyzed for SARS-CoV-2 antibodies. 

Veterinarians or veterinary technicians at Toronto Humane Society (THS) collected blood samples from cats and dogs admitted to the shelter during June 18–November 28, 2020. Any animal that did not have health and behavioral reasons for exclusion was eligible for the study, regardless of origin (surrender, seizure, stray) or known history of SARS-CoV-2 exposure. Similarly, we collected samples through Toronto Animal Services (TAS) from unowned and owned cats admitted to a low-cost neuter clinic during January 21–July 6, 2021. We centrifuged all blood samples on site and shipped serum samples to Ontario Veterinary College (Guelph, Ontario, Canada). Serum samples were frozen in aliquots until batch analysis.

We constructed ELISA assays for the detection of cat and dog IgG and IgM to SARS-CoV-2 S protein (Appendix 1). Positive controls were from a SARS-CoV-2–experimentally-infected cat and 2 dogs with high titers; negative controls were cat and dog serum samples collected before 2019.

We tested the initial 42 serum samples and a subsequent 70 samples with IgG optical density (OD) >1.4 with the surrogate virus neutralization test (sVNT; GenScript, https://www.genscript.com) to determine blocking of the interaction of the receptor-binding domain (RBD) of SARS-CoV-2 with the ACE2 receptor. Following manufacturer instructions, we interpreted inhibition >20% relative to the kit positive control as indicating the presence of neutralizing antibodies.

### Survey

We asked owners of household pets to complete an online 20-question survey concerning household demographics, the nature of the interaction with their pets, and the development of new illness in pets (Appendix 2). We also administered a questionnaire to owners of cats brought to the low-cost neuter clinic (Appendix 3). Questionnaires were not administered for unowned cats.

### Statistical Analysis

For household cats, factors associated with PCR positivity were not evaluated because of the small sample size and low prevalence. We evaluated factors associated with seropositivity by univariable analysis using χ^2^, Fisher exact, or Wilcoxon tests as appropriate for the data. We categorized neuter-clinic cats by age: cats <6 months of age were kittens and cats >6 months adults. We calculated odds ratios and 95% CI. We did not perform multivariable analysis because of limitations in sample size.

We compared differences in seropositivity among different pet cohorts with Mann-Whitney tests. We calculated correlation of ELISA OD with sVNT results using Prism version 9.3.1 (GraphPad, https://www.graphpad.com); p<0.05 was considered significant.

## Results

### PCR

We collected a total of 283 swab specimens from 65 cats, 49 dogs, and 6 ferrets: 70 nasal, 90 oral, 107 rectal and 16 fur (dorsum) samples. Samples from 5 (7.7%) cats and 1 (2.0%) dog had positive PCR results. Each N1 PCR positive result (Ct <35.99) was confirmed by amplification with E, R, or RdRp primers. For all 6 animals testing positive, the nasal swab samples were positive; oral swab samples were positive from 2 of 3 tested, and rectal swab samples were positive from 1 of 3 tested. Swab samples from an additional 10 (15%) cats, 3 (6.1%) dogs, and 3 (50%) ferrets had nonnegative results. N1 PCR Ct values for those 16 samples were 36.00–39.00. Testing of other targeted regions at additional laboratories yielded similar nonnegative results.

One cat with an initial Ct of 21.56 was retested weekly 5 times after the first positive result and had positive results during the first 3 weeks. Another cat with an initial Ct of 24.11 tested positive again 1 week later (Ct 36.19) and negative thereafter.

We derived whole-genome sequences (>99.3% coverage) from 2 positive cats. Phylogenetic analysis assigned the sequences to Pangolin lineage A.23.1 and B.1.2, which had the highest similarity to human SARS-CoV-2 sequences derived in that time period from the corresponding geographic region.

### Serology 

#### Household Pets 

We collected serum samples from 59 dogs and 48 cats from 77 households and 1 animal shelter (from recently surrendered cats). Median number of samples per household was 1 (range 1–4). We collected 7 samples from the humane society; those 7 samples were excluded from risk factor analysis because of the potential clustering effect and the lack of metadata about these animals. Dogs were a median of 5 years of age (range 5 months–14 years of age), and cats were a median of 6 years of age (range 1–19 years of age).

Seropositivity for IgG and IgM was 42%–62% using >3 SD above the mean of the negative control samples as a cutoff and 25%–48% at >6 SD (Table 1). At >6 SD, all IgM positive dogs were also IgG positive, whereas 12/48 (25%) cats were IgG positive but IgM negative.

**Table 1:** Serology results from dogs and cats whose owners had received a diagnosis of SARS-CoV-2 infection or had symptoms compatible with COVID-19 in the previous 3 weeks, Ontario, Canada*

Test result	IgG	IgM	IgG and IgM	IgG or IgM
Dogs, n = 59				
>3 SD	26 (44)	26 (44)	21 (36)	31 (53)
>6 SD	22 (37)	16 (27)	16 (27)	24 (41)
Cats, n = 48				
>3 SD	29 (60)	29 (60)	22 (46)	35 (73)
>6 SD	23 (48)	13 (27)	11 (23)	25 (52)

*Values are no. (%). Results were >3 or >6 SD above the mean result for negative controls.

For statistical analysis, we defined seropositivity as >3 SD for IgG, IgM, or both. We observed a significant association between seropositivity and owner-reported onset of new respiratory disease in dogs at the time of the owner’s infection (p = 0.04), but not in cats (Table 2). Association of seropositivity and owner-reported new onset of clinical signs (respiratory, gastrointestinal, or systemic signs such as lethargy) approached significance in dogs (p = 0.06).

**Table 2:** Association of seropositivity for SARS-CoV-2 in pets with household risk factors and development of new illness, Ontario, Canada*

Variable	Dogs, n = 59				Cats, n = 48		
	Seropositive^*^	Seronegative	p value		Seropositive	Seronegative	p value
Kissed by owner	16/25 (64)	16/27 (59)	0.73		16/27 (59)	3/13 (30)	0.15
Licked hands/face of owner	19/25 (64)	22/25 (81)	0.63		13/27 (48)	3/13 (30)	0.46
Slept in/on bed	17/24 (68)	15/27 (56)	0.36		23/27 (85)	5/10 (50)	0.04
New respiratory signs	9/29 (31)	2/27 (7.4)	0.04		8/29 (28)	2/10 (20)	1.00
New clinical signs	12/29 (41)	5/27 (19)	0.06		12/29 (41)	2/10 (20)	0.28

*Seropositivity is defined by IgG, IgM or both against viral S protein. Results were positive if optical density is >3 SD above the mean of negative controls.

Not all risk factor data were available for all animals. Univariable risk factor analysis did not identify risk factors for dogs, but sleeping in the owner’s bed was a risk factor for seropositivity in cats (OR 5.8, 95% CI 1.1–29.4) We determined no effect from the presence of multiple pets in the household (dogs p = 0.33, cats p = 0.70) or the number of persons with confirmed (dogs p = 0.77, cats p = 0.64) or confirmed and suspected (dogs p = 0.92, cats p = 0.47) COVID-19. We did not see an association between time the animal typically spent per day with the infected owner for either dogs (p = 0.71) or cats (p = 0.53).

When we defined seropositivity as >6 SD above the mean of negative controls, we saw no significant association between seropositivity and owner-reported onset of new respiratory disease in the pet at the time of the owner’s infection for dogs (Table 3). However, we observed a significant association of seropositivity and owner-reported new onset of clinical respiratory, gastrointestinal, or systemic signs such as lethargy in the pet. We found the same association in cats.

**Table 3:** Association of seropositivity for SARS-CoV-2 in pets with household risk factors and development of new illness, Ontario, Canada*

Variable	Dogs, n = 59				Cats, n = 48		
	Seropositive	Seronegative	p value		Seropositive	Seronegative	p value
Multiple pets	9/24 (38)	15/19 (44)	0.79		15/27 (56)	12/19 (63)	0.61
Kissed by owner	13/20 (65)	19/32 (59)	0.69		11/19 (58)	8/18 (44)	0.52
Licked hands/face of owner	16/20 (80)	25/32 (78)	1.00		10/19 (53)	6/18 (33)	0.32
Slept in/on bed	13/20 (65)	19/32 (59)	0.69		17/19 (76)	11/18 (61)	0.06
New respiratory signs	7/23 (30)	4/33 (12)	0.17		8/21 (38)	2/18 (11)	0.07
New clinical signs	11/23 (48)	6/33 (18)	0.018		12/21 (57)	2/18 (11)	0.006

*Seropositivity is defined by IgG, IgM or both against viral S protein. Results were positive if optical density is >6 SD above the mean of negative controls.

Univariable risk factor analysis did not identify an association of seropositivity with risk factors (Table 3). We saw no association between time the animal typically spent per day with the infected owner for either dogs (p = 0.73) or cats (p = 0.35). However, cats that spent <2 hours per day with their owner were significantly less likely to be seropositive (1/7 [16%] vs. 18/30 [67%]; p = 0.04). We did not see the same result for dogs (p = 0.51). We saw no effect from the presence of multiple pets in the household (dogs p = 0.61, cats p = 0.69) or the number of persons per household with confirmed (dogs p = 0.83, cats p = 0.74) or confirmed or suspected (dogs p = 0.84, cats p = 0.82) COVID-19. Overall, >1 animal was seropositive in 3 (16%) of the 19 households where >1 animal was sampled: 2 households in which 2 dogs were seropositive and 1 in which a dog and cat were seropositive.

We performed sVNT on 53 samples from household pets. Of those, 30/41 (76%) that were positive for IgG and/or IgM (6 SD) were also positive on sVNT compared with 0/12 IgG/IgM negative samples (p<0.0001). Despite the smaller sample size, we repeated risk factor analysis using the samples tested by sVNT. For dogs, licking hands or face of owners was associated with seropositivity (OR 10.5 95% CI 1.5–73; p = 0.017). In addition, we noticed an association between positivity and dogs spending 19–24 hours with owners (OR 13.3, 95% CI 1.3–135; p = 0.033). For cats, the association between positivity and being kissed by owners was significant (OR 18.7, 95% CI 1.6–223; p = 0.020).

#### Neuter-Clinic Cats

**Figure 1:**
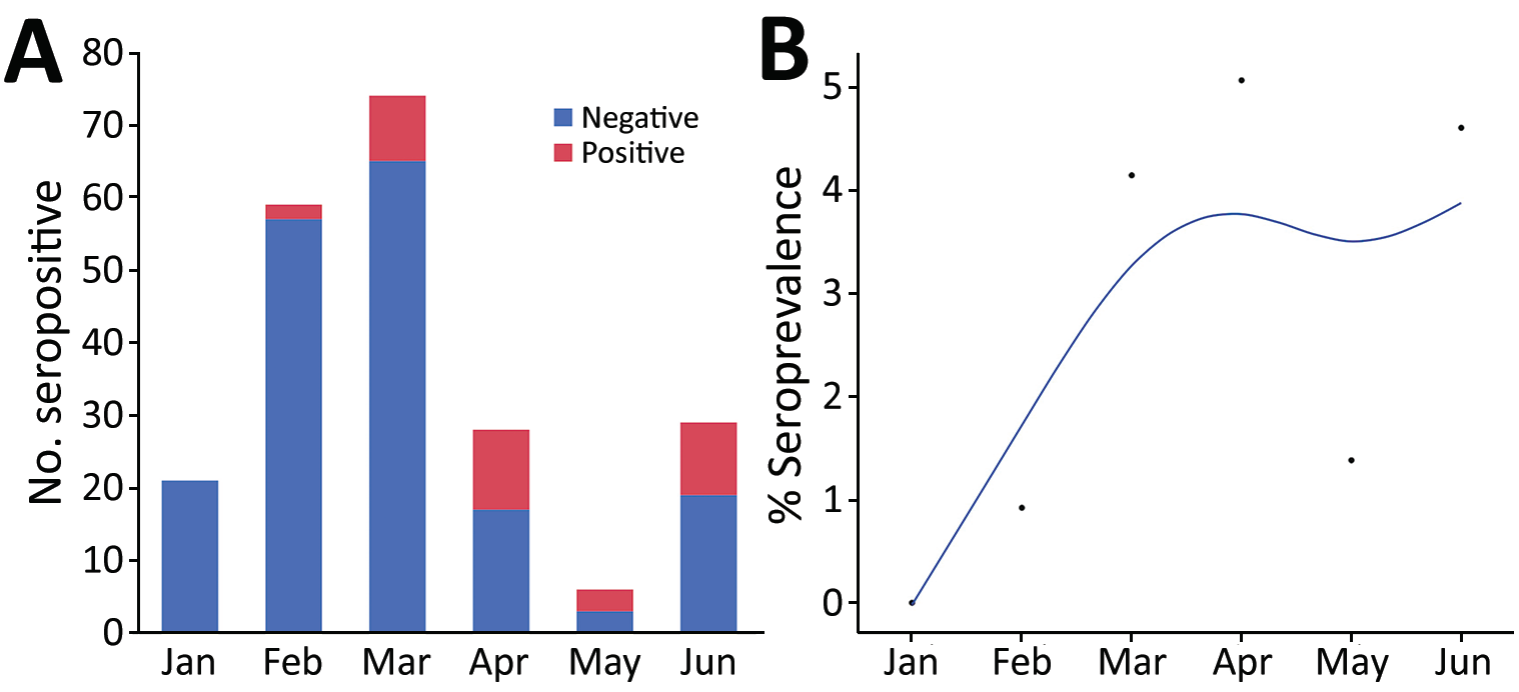
Seropositivity for SARS-CoV-2 in cats brought for care to a low-cost spay/neuter clinic during January – June 2021, Ontario, Canada. A) Test results for 221 cats shown by month. B) Positivity rate per month. The points indicate the proportion of positive test results among all test results over time. Blue line indicates the smoothed rate of seropositivity. The association between month and the change in seropositivity was significant (p<0.0001).

We collected serum samples from 221 cats during January–June 2021. Full animal information and history were not available for all cats. The median age of the 184 (83%) cats for which age was reported or estimated was 1.5 years (interquartile range 3.25 years). We classified 32/184 (17%) cats as kittens and 152 (83%) as adults (Table 4). COVID contact status was known for 103 cats. We detected S IgG (>6 SD) in 35/221 (16%) cats. Monthly seropositivity rate was 0%–40%; we identified a significant association between month and seropositivity (p<0.0001) (Figure 1).

**Table 4:** Characteristics of 221 cats at a neuter clinic tested for the presence of SARS-CoV-2 serum antibodies and univariable analysis results, Ontario, Canada

Characteristic	Seropositive, no. (%)	p value
Categorical age		0.12
Kitten	2/32 (6.3)	
Adult	27/152 (18)	
Sex		1.0
M	16/106 (15)	
F	12/78 (15)	
Not reported	7/37 (19)	
Animal source		0.01
Household pet	7/93 (8)	
Shelter/rescue/foster	23/102 (23)	
Feral	5/26 (19)	
Exposure to person with COVID		0.59
Yes	2/13 (15)	
No	6/90 (6.7)	
Unknown or declined to answer	27/118 (23)	

Univariable analyses were performed excluding animals whose exposure to persons with COVID-19 was unknown (Table 4). We identified animal source as a risk factor for seropositivity. Compared with cats originating from households, cats that were in a shelter, rescue or foster facility cats were 3.6 times as likely to be seropositive (95% CI 1.5–8.8; p = 0.005). We found no significant difference between feral and household cats or feral and shelter/rescue/foster cats.

#### Humane Society Animals

Of 67 cat and 8 dog samples from THS, 7/75 (9.3%) overall and 7/67 (10%) of cat samples were seropositive (>6 SD). We did not perform risk factor analysis because limited metadata were available.

### Correlation of ELISA with sVNT

**Figure 2:**
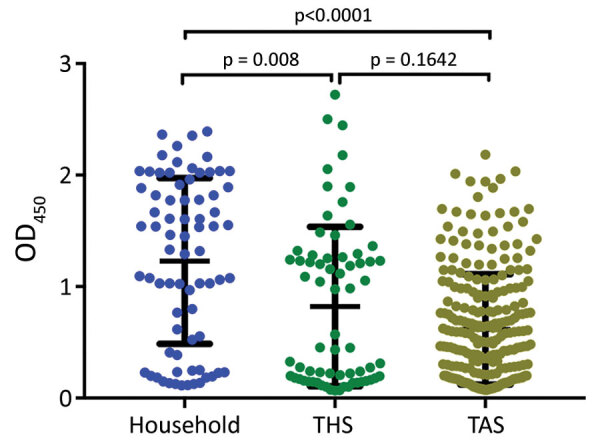
Mean serum SARS-CoV-2 spike protein IgG as measured by ELISA for samples from household cats, from cats in a shelter (THS), and from cats brought to a spay/neuter clinic for care (TAS), Ontario, Canada. The mean and SD are indicated. Differences were significant for household vs. shelter cats and household vs. clinic cats, but not for shelter vs. clinic cats. OD_450_, optical density at 450 nm; THS, Toronto Humane Society; TAS, Toronto Animal Services.

We identified a significant difference in the mean OD between household samples and those from both THS and TAS. Differences between THS and TAS were not significant (Figure 2).

**Figure 3:**
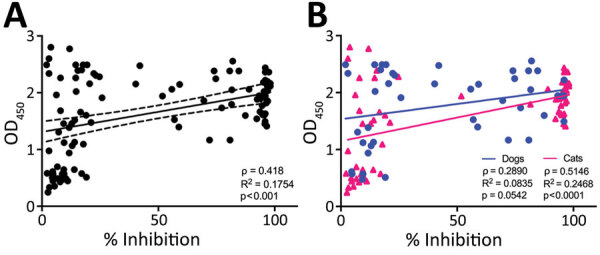
Results of IgG ELISA in relation to percentage inhibition of binding of the SARS-CoV-2 receptor binding domain (RBD) to the ACE2 receptor in cat and dog serum samples measured with a surrogate virus neutralization assay, Ontario, Canada. A) Surrogate virus neutralization test results correlated with IgG ELISA results. B) Percentage of inhibition for dog (blue circles) and cat (pink triangles) samples. The solid line shows correlation and dashed lines 95% CI. Correlation is higher for cat than dog samples. OD_450_, optical density at 450 nm.

In addition to ELISA testing, we also assessed a subset of 112 serum samples (53 household and 59 from shelter and spay/neuter clinic) with the sVNT. We found a significant correlation between the ELISA OD and neutralization of virus binding (ρ = 0.4188, 95% CI 0.2529–0.5608; p<0.0001) (Figure 3, panel A). The correlation between ELISA and sVNT results was higher for cats than dogs (Figure 3, panel B).

## Discussion

Our findings suggest that transmission of SARS-CoV-2 from infected humans to their pets as indicated by seroconversion is common. PCR-based detection of SARS-CoV-2 in pets was uncommon within 3 weeks from owners being symptomatic or having a diagnosis of COVID-19, which may reflect genuine brevity of infection in pets, as noted experimentally in cats (*12*).Other factors are variations in time intervals between owner infection and pet sampling and the challenge of obtaining representative samples from the nose in cats (*12*). Other studies of infection of cats from households of persons with COVID-19 had similarly low PCR-based prevalence (*16*,*25*–*28*). The timeframe required for owners to be diagnosed, contact the study team, and arrange a household visit likely resulted in false negative PCR results from samples being collected too late relative to onset of infection. The definition of COVID-19 symptoms and access to PCR testing for sick persons was limited early in the pandemic, and it is possible that pets in this study were infected concurrently or immediately after their owners but swabbed only after they had eliminated infection. Kittens 4–5 months old experimentally infected with 1 × 10^6^ TCID_50_ of SARS-CoV-2 intranasally and orally had detectable viral RNA for 10 days in nasopharyngeal swabs, 7 days in oropharyngeal swabs, and 14 days in rectal swabs, but such high viral challenge may not simulate typical human–cat household interactions (*12*). Subtle pulmonary lesions and viral RNA detectable until 6 days postinfection in experimentally infected cats suggest that, even with high viral inoculates, cats rarely get sick and can clear infection relatively quickly (*29*).

Longitudinal samples were rarely available; however, serial sampling for 1 cat revealed prolonged PCR positivity. That cat had chronic upper respiratory disease; whether the condition played a role in the prolonged PCR positivity is unclear. Despite the duration of PCR positivity, it is unlikely that the cat was infectious because the relatively high PCR Ct values would be consistent with low-level shedding of viral nucleic acids. Similar prolonged PCR positivity has been reported for a cat exposed in a retirement home (*30*) and for tigers and lions in zoos (*31*). More data regarding the duration of positivity in naturally infected dogs and cats, and whether infectious virus is shed, are needed.

Seroprevalence was much higher than PCR positivity. We expected this finding because serologic data represent historical exposure and there is not a need to sample animals within a narrow infection window. Seroprevalence detected in other studies was 0.4%–30% or higher; in most instances such variability could be attributed to the extent of pets’ exposure to infected humans (*6*,*9*,*32*–*34*).

Without broadly accepted definitions, the parameters and interpretation of serologic assays for SARS-CoV-2 vary widely (*13*,*28*,*35*–*37*). We designed traditional ELISAs detecting IgG and IgM for S protein. We used a range of negative serum samples from before 2019, as well as serum from cats with feline infectious peritonitis caused by enteric α coronavirus. The negative controls yielded consistently low ODs for S protein IgG and IgM; we interpreted results from exposed animals at 3 SD and 6 SD above the mean of the least diluted negative controls to enable comparison with other studies (*12*,*13*). A relatively high proportion of dogs and cats had antibodies to S protein, which could indicate infection or exposure. Results of the sVNT, most likely to reflect infection, correlated with S protein ELISA results in this and other studies (*38*). Some serum samples had high S antibodies despite lack of neutralization; this pattern could indicate exposure rather than infection or postinfection persistence of antibodies broadly reactive with S protein but not neutralizing RBD binding. The cause of the discrepant results cannot be determined from samples collected at a single time point that was potentially days or weeks postexposure. Furthermore, development of antibodies to SARS-CoV-2 is affected by host age, immunocompetence, and comorbidities, which could not be controlled in this surveillance study (*36*); even experimentally infected young cats had inconsistent antibody responses (*12*).

Risk factor analyses identified plausible associations presumably linked to the duration and closeness of human–animal contact. Limited risk factor information for dogs and cats has been reported (*16*, *28**,**37*,*39*); however, association of seropositivity and proximity or sleeping with infected owners has been reported for dogs (*16*) and in a study where canine and feline data were combined (*40*). In our study, the same risk factors were not identified when using different serologic cutoffs or tests, which was likely a result of small sample sizes.

The substantially higher seroprevalence in cats exposed to infected persons gives more credence to the seropositivity data. Yet, the prevalence of seropositivity was still moderately high in cats with no known exposure to infected people. The lack of metadata makes this challenging to interpret, because it is possible that cats from the humane society or neuter clinic had previously been exposed to infected humans (*28*).

PCR positivity rate was too low for robust comparison of sample sites. However, all positive animals had positive nasal swab specimens, despite the challenges that can be encountered collecting good nasal swabs, especially from cats. Adding oral, rectal, or fur swab specimens did not increase diagnostic yield. Further study of sampling sites under field conditions would identify sampling approaches that maximize diagnostic yield while minimizing the number of sites that must be sampled. These data are preliminary but support the importance of collecting nasal swab specimens as part of or all of the sample set.

Our study’s first limitation was sample size; enrollment was hampered by low human COVID-19 infection rates in the study region throughout the main sampling times and by difficulties identifying exposed households in an appropriate timeframe. Lack of a coordinated One Health approach concurrently investigating human and animal exposures was a problem; local or provincial public health agencies had little interest in leading research or performing a joint study. The timing of sampling also affected PCR results. More complete validation of the specificity of serologic assays with a samples from animals with diverse other infectious and inflammatory conditions remains to be done. Ideally, the timeframe for sampling would have been more condensed to focus testing on animals whose owners were more recently infected (e.g., 1–2 weeks after the onset of the owner’s infection).

These data indicate relatively common transmission of SARS-CoV-2 from humans to animals and that certain human–animal contacts (e.g., kissing the pet, pet sleeping on the bed) appear to increase the risk. We inferred that infections in dogs and cats reflect direct transmission from humans to animals, given the pandemic nature of this virus in humans and limited contact of most household pets with other animals (*41*). Intra-household transmission cannot be ruled out as a cause of some infections; however, multiple seropositive animals were only identified in 3/19 (16%) households where multiple animals were tested. We did not specifically investigate whether this relates to differences in individual animal susceptibility or animal–owner contact.

The relevance of human–pet transmission of SARS-CoV-2 needs further study. We observed an association between infection and clinical disease in both dogs and cats; in most cases, disease was very mild and self-limiting. Clinical data from this study are consistent with other studies indicating limited overall health risk to otherwise healthy dogs and cats (*17*,*18*,*42*). The zoonotic risk posed by dogs is probably low based on the lower infection rate and lack of evidence of transmission experimentally (*43*). Risk is probably higher for cats; cat–cat transmission has been identified, but the actual risk for cat–human transmission is unknown (*44*). Our findings support the occurrence of human–dog and human–cat transmission and highlight the need for further study of the animal and human health consequences of spillback of this zoonotic pathogen into animals.

Appendix 1 Additional information about SARS-CoV-2 infection, seropositivity, and illness in cats and dogs.

Appendix 2 Questionnaire used in surveillance of SARS-CoV-2 infection, seropositivity, and illness in household pets.

Appendix 3 Questionnaire used in surveillance of SARS-CoV-2 infection, seropositivity, and illness in cats and dogs in an animal shelter.
