# Supplementary material for: Risk Factors for SARS-CoV-2 Infection and Illness in Cats and Dogs
Source: Emerg Infect Dis. 2022 Jun;28(6):1154–62. doi: 10.3201/eid2806.220423 (PMC9155877; doi:10.3201/eid2806.220423)
Supplement: Appendix 1 — Additional information about SARS-CoV-2 infection, seropositivity, and illness in cats and dogs. [file 22-0423-Techapp-s1.pdf]

Article DOI: https://doi.org/10.3201/eid2806.220423

Risk Factors for SARS-CoV-2 Infection and Illness in Cats and Dogs

Appendix 1

Materials and Methods

PCR and Whole-Genome Sequencing

RNA was reverse transcribed and SARS-CoV-2 cDNA was amplified by qPCR (AgPath-ID, Applied Biosystems) with primers to the viral N1 gene 3′ GAC CCC AAA ATC AGC GAA AT 5′ and 3′ CAG ATT CAA CTG GCA G 5′, and detected with the probe 3′ ACC CCG CAT TAC GTT TGG 5′ labeled with fluorescein amidite (FAM). Viral RNA was also amplified with primers to the N2 gene 3′ TTA CAA ACA TTG GCC GCA AA 5′ and 3′ TTC TTC GTA ATG TCG CGC 5′ and detected with the FAM-labelled probe 3′ ACA ATT TGC CCC CAG CGC TTC 5′. Primers and probe were based on the SARS-CoV-2 sequence GenBank MT226610.1. Host cellular RNA extracted from swabs was amplified with primers 3′ CAA TTT CCA ATG CCC TCA AYT T 5′ and 3′ CAC ATC GTA TGG GCC TCT TAT T 5′ directed against the ribonuclease P/MRP subunit p30 (RPP30) and detected with an Affinity Plus probe TC+T+A+GTG+C+T+GC. All oligonucleotides were obtained from Integrated DNA Technologies (Coralville, IA). Nucleic acids were amplified and quantified in a Light Cycler 480 (Roche, Mississauga, ON). The positive control consisted of inactivated SARS-CoV-2/Canada/VIDO-01/2020, kindly provided by the National Microbiology Laboratory (Winnipeg, MB). Only samples with Ct values <35.99 for RP30 were considered to yield interpretable viral PCR results. Samples with viral Ct values <35.99 were considered positive, samples with Ct values of 36–39.00 were considered non-negative.

All samples with Ct <39 were submitted for additional amplifications with primers to the viral E (envelope) and RdRp (RNA-dependent RNA polymerase) genes at the Animal Health Laboratory (Guelph, ON) and the Canadian Food Inspection Agency (CFIA) at the National Microbiology Laboratory (NML), Winnipeg, MB. Whole-genome sequencing and analysis was

performed at the NML or the British Columbia Centre for Disease Control Public Health Laboratory using an amplicon method (Artic V3 or Freed 1200bp) on an Illumina instrument. Sequences were analyzed alongside human SARS-CoV-2 sequences from the household or the region to assess virus relatedness.

ELISA

Enzyme-linked immunosorbent assays (ELISA) for the detection of cat and dog IgG and IgM antibodies to SARS-CoV-2 S protein were constructed. Briefly, adsorption immunoassay plates (96-well, ThermoFisher, Mississauga, ON) were coated overnight at 4º C with 2 µg/mL of His-tagged SARS-CoV-2 S1 (GenScript, Piscataway, NJ). Next day, wells were washed 3×, blocked with 3% skim milk in Tris buffer for 1 hour, and washed 3×; then 60 µL of five 3-fold dilutions (1:100, 1:300, 1:900, 1:2,700, and 1:8,100) of each serum sample was added. Plates were incubated for 2 hours, washed 3×, and secondary antibodies conjugated to horseradish peroxidase (HRP) and diluted 1:5,000 were added for 1 hour. Wells were washed 3×, and HRP activity was visualized by adding trimethyl benzidine (TMB) substrate. Reactions were stopped with sulfuric acid, and optical density (OD) at 450 nm was read. Secondary antibodies consisted of goat anti-dog IgG, goat anti-dog IgM, goat anti-cat IgG and goat anti-cat IgM (all from Abcam, Waltham, MA). Control samples consisted of serum from a SARS-CoV-2 experimentally-infected cat (kindly provided by Y. Kawaoka, Madison, WI; positive feline control, used at 1:5,000 in ELISA), 3 different batches of pooled cat serum from 2016 or 2017, 2 serum samples from cats with feline infectious peritonitis, 1 serum sample from a cat with osteomyelitis and hyperglobulinemia (Appendix. Figure 1). Control dog samples were from 2017, 2018, and 2019 (negative controls, Appendix Figure 2). One dog sample with high OD was used across all ELISA plates as a positive control (Appendix Figure 3). Each ELISA plate included 16 wells that were not coated with recombinant protein (blank), 5 replicate 1:100 dilutions of species-specific negative control samples, and five 3-fold dilutions of the positive control and test samples, starting at a 1:100 dilution.

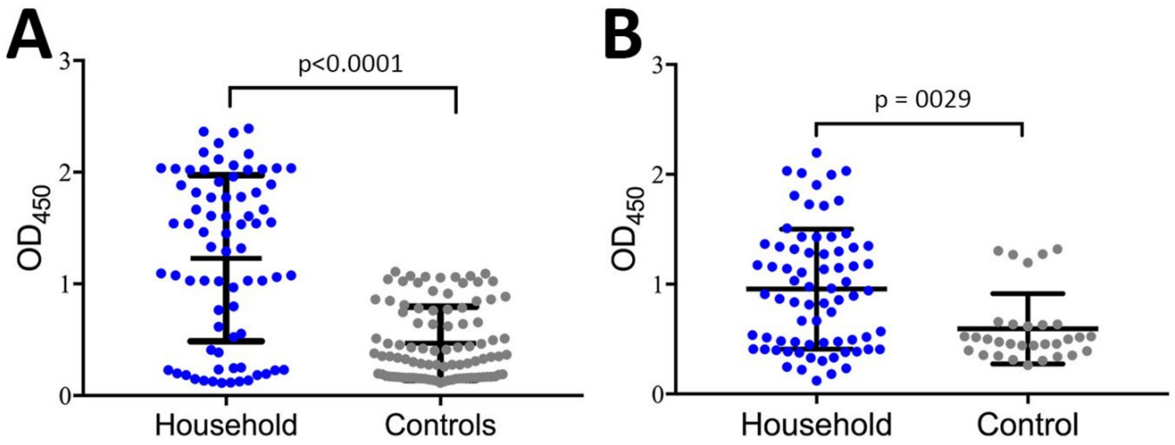

Appendix Figure 1. Optical density for IgG and IgM to S protein in household cat samples relative to control samples.

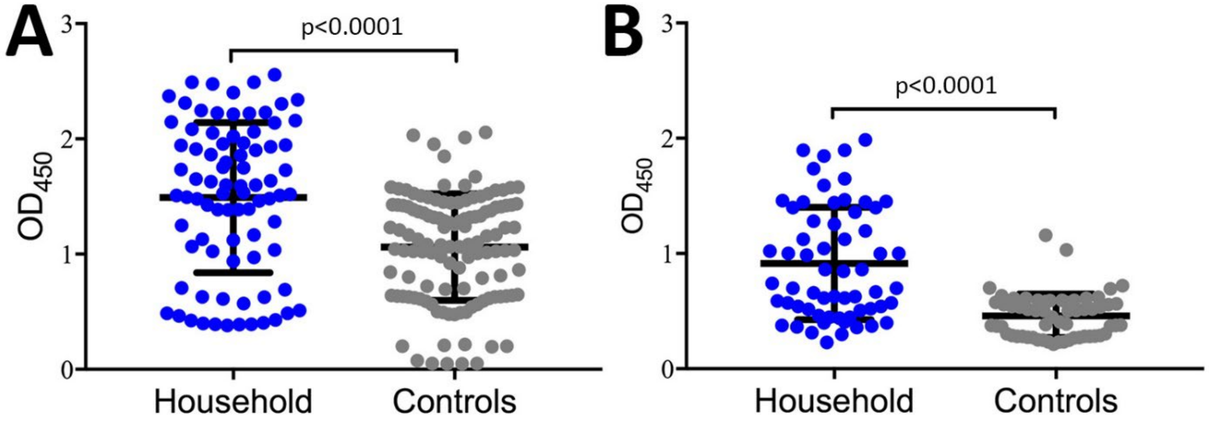

Appendix Figure 2. Optical density for IgG and IgM to S protein in household dog samples relative to control samples.

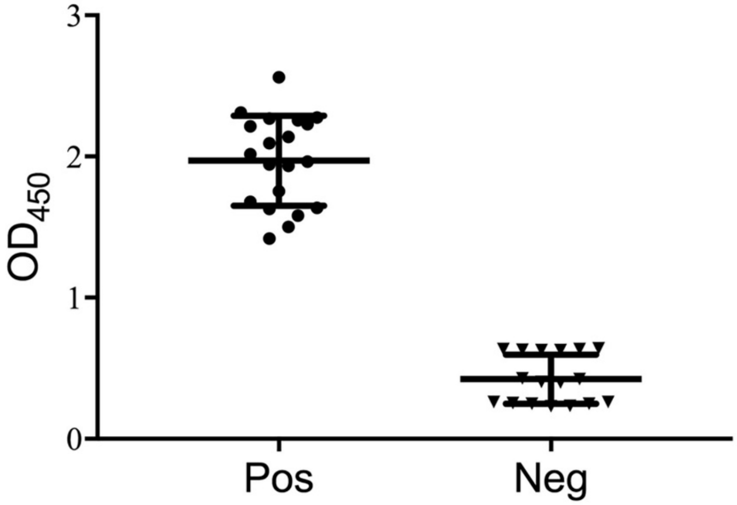

Appendix Figure 3. Repeatability of optical density for a positive dog sample relative to negative samples, IgG to S protein.
