# Supplementary material for: Risk Factors for SARS-CoV-2 Infection and Illness in Cats and Dogs
Source: Emerg Infect Dis. 2022 Jun;28(6):1154–62. doi: 10.3201/eid2806.220423 (PMC9155877; doi:10.3201/eid2806.220423)
Supplement: Appendix 2 — Questionnaire used in surveillance of SARS-CoV-2 infection, seropositivity, and illness in household pets. [file 22-0423-Techapp-s2.pdf]

Article DOI: https://doi.org/10.3201/eid2806.220423

Risk Factors for SARS-CoV-2 Infection and Illness in Cats and Dogs

Appendix 2

Online survey

A survey given to owners of household pets is shown on the following pages.

COVID serology

Start of Block: Default Question Block

Q1 **Evaluation of antibodies against SARS-CoV-2, the cause of COVID-19, in pets exposed to infected people** This study will evaluate whether or how commonly SARS-CoV-2 can be found in animals. You are being invited to participate because you have indicated that one or more people in your household had COVID-19. The study aims to help us better understand transmission of this virus between people and their pets. Participants must be 18 years of age or older. This study is funded by the Ontario Animal Health Network.
If you agree to take part in this study, you will be asked to complete a short (5 minute) survey. You will be asked to provide your name, phone number or email address, information about COVID-19 in your household and pet contact information. You will not be asked to identify specific people that are known or suspected to have COVID-19. Information from this survey will help understand COVID-19 in animals and transmission of the virus between people and animals. This survey is voluntary and all identifying information will be treated as confidential. Only Drs. Weese and Bienzle will have access to the data. No identifying or individual results will be released and identifying information will be stored on an encrypted device. Although there is a risk that you may be concerned about how your responses will be evaluated or about providing information about COVID-19 diagnoses, there are no right or wrong answers. You will not be asked any details about anyone's medical status beyond whether people in contact with the pet are known or suspected to be infected, and this information will be confidential. You will not be contacted about your survey results or participation, but will be informed of the testing results of your pet.
By entering this survey, you indicate that you have read the information provided and consent to participate. There are no direct benefits to you, but results will improve our understanding SARS-CoV-2. You may choose to skip any question(s) you do not want to answer, apart from your name and one form of contact. You can stop completing the survey at any time. If you wish to have your answers removed or if you have any questions, you may contact Dr. Weese at 519-824-4120 ext 54064 or jsweese@uoguelph.ca. After Mar 1, 2021, we will not be able to remove data.
A summary of results will be available at http://www.wormsandgermsblog.com upon completion of the study and be part of a scientific paper. Data will be retained on a secure drive until publication of results, then survey results will be destroyed. We encourage you to print a copy of this consent page.
You do not waive any legal rights by agreeing to take part in this study. This study has been reviewed by the University of Guelph Research Ethics Board for compliance with federal guidelines for research involving human participants. If you have any questions about your rights as a research participant in this study (REB#20-04-002) please contact: Manager,

Research Ethics, University of Guelph, reb@uoguelph.ca; 519-824-4120 ext 56606.By continuing on to the survey, you are indicating your consent to participate in this survey.

Q2 What is your name?

____

Q3 What is the best phone number or email address to reach you at to provide you with test results?

____

Q4 At which veterinary clinic was your pet tested?

____

Q5 How many people currently live in your household?

____

Q6 How many people in your household were diagnosed with COVID-19?

____

Q7 How many were not tested but were told by a healthcare provider or public health personnel that they likely had COVID-19?

____

Q8 Approximately what date was COVID-19 first diagnosed or suspected in the household?

____

End of Block: Default Question Block

Start of Block: Block 2

Q74 Do you have one or more dogs that were tested?

○ Yes (1)

○ No (2)

Skip To: Q28 If Do you have one or more dogs that were tested? = No

Q11 Dog's name (if you have more than one dog, pick one and we'll ask about the others later)

____

Q12 During the time that someone in the household had COVID-19, to the best of your recollection, which of the following occurred with this dog? (Check all that apply)

- ▢ Went on walks on a leash (1)
- ▢ Went to a dog park on a leash (2)
- ▢ Went to an off-leash park (3)
- ▢ Was off your property without supervision (4)
- ▢ Spent time unsupervised in a fenced yard or tied up (5)
- ▢ Visited a veterinary clinic (6)
- ▢ Visited a human healthcare facility (7)
- ▢ Went to a groomer (8)
- ▢ Went to a kennel (9)

Q13 When people in your household had COVID-19, which of the following likely occurred? (please check all that apply)

- ☐ Slept in or on the bed of an infected person (1)
- ☐ Licked the face of an infected person (2)
- ☐ Licked the hands of an infected person (3)
- ☐ Was kissed by an infected person (4)
- ☐ Sat or the lap of, or beside, an infected person (5)

Q14 When people in your household had COVID-19, approximately how much time did this dog spend in the same room as an infected person on an average day?

- ○ Less than 2 hours (1)
- ○ 2-6 hours (2)
- ○ 7-12 hours (3)
- ○ 13-18 hours (4)
- ○ 19-24 hours (5)

Q15 Around the time that people in the household had COVID-19, did this dog have any new occurrences of the following? (please check all that apply)

- ▢ Cough (1)
- ▢ Difficulty breathing (2)
- ▢ Vomiting (3)
- ▢ Diarrhea (4)
- ▢ Decreased appetite (5)
- ▢ Decreased energy (6)

Q16 Do you have another dog?

- ○ Yes (1)
- ○ No (2)

Skip To: Q28 If Do you have another dog? = No

Q17 Dog #2's name

____

Q18 During the time that someone in the household had COVID-19, to the best of your recollection, which of the following occurred with this dog? (Check all that apply)

- ☐ Went on walks on a leash (1)
- ☐ Went to a dog park on a leash (2)
- ☐ Went to an off-leash park (3)
- ☐ Was off your property without supervision (4)
- ☐ Spent time unsupervised in a fenced yard or tied up (5)
- ☐ Visited a veterinary clinic (6)
- ☐ Visited a human healthcare facility (7)
- ☐ Went to a groomer (8)
- ☐ Went to a kennel (9)

Q19 When people in your household had COVID-19, which of the following likely occurred? (please check all that apply)

- ▢ Slept in or on the bed of an infected person (1)
- ▢ Licked the face of an infected perso (2)
- ▢ Licked the hands of an infected person (3)
- ▢ Was kissed by an infected person (4)
- ▢ Sat or the lap of, or beside, an infected person (5)

Q20 When people in your household had COVID-19, approximately how much time did this dog spend in the same room as an infected person on an average day?

- ○ Less than 2 hours (1)
- ○ 2-6 hours (2)
- ○ 7-12 hours (3)
- ○ 13-18 hours (4)
- ○ 19-24 hours (5)

Q21 Around the time that people in the household had COVID-19, did this dog have any new occurrences of the following? (please check all that apply)

- ☐ Cough (1)
- ☐ Difficulty breathing (2)
- ☐ Vomiting (3)
- ☐ Diarrhea (4)
- ☐ Decreased appetite (5)
- ☐ Decreased energy (6)

Q22 Do you have another dog?

- ○ Yes (1)
- ○ No (2)

Skip To: Q28 If Do you have another dog? = No

Q23 Dog #3's name

____

Q24 During the time that someone in the household had COVID-19, to the best of your recollection, which of the following occurred with this dog? (Check all that apply)

- ▢ Went on walks on a leash (1)
- ▢ Went to a dog park on a leash (2)
- ▢ Went to an off-leash park (3)
- ▢ Was off your property without supervision (4)
- ▢ Spent time unsupervised in a fenced yard or tied up (5)
- ▢ Visited a veterinary clinic (6)
- ▢ Visited a human healthcare facility (7)
- ▢ Went to a groomer (8)
- ▢ Went to a kennel (9)

Q25 When people in your household had COVID-19, which of the following likely occurred? (please check all that apply)

- ☐ Slept in or on the bed of an infected person (1)
- ☐ Licked the face of an infected perso (2)
- ☐ Licked the hands of an infected person (3)
- ☐ Was kissed by an infected person (4)
- ☐ Sat or the lap of, or beside, an infected person (5)

Q26 When people in your household had COVID-19, approximately how much time did this dog spend in the same room as an infected person on an average day?

- ○ Less than 2 hours (1)
- ○ 2-6 hours (2)
- ○ 7-12 hours (3)
- ○ 13-18 hours (4)
- ○ 19-24 hours (5)

Q27 Around the time that people in the household had COVID-19, did this dog have any new occurrences of the following? (please check all that apply)

- ☐ Cough (1)
- ☐ Difficulty breathing (2)
- ☐ Vomiting (3)
- ☐ Diarrhea (4)
- ☐ Decreased appetite (5)
- ☐ Decreased energy (6)

Q28 Do you also have a cat that was tested?

- ○ Yes (1)
- ○ No (2)

Skip To: End of Survey If Do you also have a cat that was tested? = No

End of Block: Block 2

Start of Block: Block 4

Q29 Cat's name (if you have more than one, pick one and we'll ask about the others next)

____

Q30 What best describes your cat

- ○ Indoor exclusively (1)
- ○ Mainly indoor with some outdoor access (2)
- ○ Spends large amounts of time both indoors and outdoors (3)
- ○ Mainly outdoor (4)
- ○ Outdoor exclusively (5)

Q31 When people in your household had COVID-19, which of the following likely occurred? (please check all that apply)

- ▢ Slept on/in the bed of an infected person (1)
- ▢ Licked the face or hands of an infected person (2)
- ▢ Was kissed by an infected person (3)
- ▢ Sat or the lap of, or beside, an infected person (4)

Q34 When people in the household had COVID-19, approximately how much time did this cat spend in the room of an infected person on an average day?

- ○ Less than 2 hours (1)
- ○ 2-6 hours (2)
- ○ 7-12 hours (3)
- ○ 13-18 hours (4)
- ○ 19-24 hours (5)

Q33 Around the time that people in the household had COVID-19, did this cat have any new occurrences of the following? (please check all that apply)

- ☐ Cough (1)
- ☐ Difficulty breathing (2)
- ☐ Vomiting (3)
- ☐ Diarrhea (4)
- ☐ Decreased appetite (5)
- ☐ Decreased energy (6)

Q75 Do you have another cat that was tested?

- ○ Yes (1)
- ○ No (2)

Skip To: End of Survey If Do you have another cat that was tested? = No

Q43 Cat #2's name

____

Q44 What best describes your cat

- ◯ Indoor exclusively (1)
- ◯ Mainly indoor with some outdoor access (2)
- ◯ Spends large amounts of time both indoors and outdoors (3)
- ◯ Mainly outdoor (4)
- ◯ Outdoor exclusively (5)

Q45 When people in your household had COVID-19, which of the following likely occurred? (please check all that apply)

- ▢ Slept on/in the bed of an infected person (1)
- ▢ Licked the face or hands of an infected person (2)
- ▢ Was kissed by an infected person (3)
- ▢ Sat or the lap of, or beside, an infected person (4)

Q46 When people in the household had COVID-19, approximately how much time did this cat spend in the room of an infected person on an average day?

- ◯ Less than 2 hours (1)
- ◯ 2-6 hours (2)
- ◯ 7-12 hours (3)
- ◯ 13-18 hours (4)
- ◯ 19-24 hours (5)

Q47 When people in the household had COVID-19, approximately how much time did this cat spend in the room of an infected person on an average day?

- ○ Less than 2 hours (1)
- ○ 2-6 hours (2)
- ○ 7-12 hours (3)
- ○ 13-18 hours (4)
- ○ 19-24 hours (5)

Q48 Around the time that people in the household had COVID-19, did this dog have any new occurrences of the following? (please check all that apply)

- ▢ Cough (1)
- ▢ Difficulty breathing (2)
- ▢ Vomiting (3)
- ▢ Diarrhea (4)
- ▢ Decreased appetite (5)
- ▢ Decreased energy (6)

Q78 Do you have another cat that was tested?

- ◯ Yes (1)
- ◯ No (2)

Skip To: End of Survey If Do you have another cat that was tested? = No

Q49 Cat #3's name

____

Q50 What best describes your cat

- ◯ Indoor exclusively (1)
- ◯ Mainly indoor with some outdoor access (2)
- ◯ Spends large amounts of time both indoors and outdoors (3)
- ◯ Mainly outdoor (4)
- ◯ Outdoor exclusively (5)

Q51 When people in your household had COVID-19, which of the following likely occurred? (please check all that apply)

- ▢ Slept on/in the bed of an infected person (1)
- ▢ Licked the face or hands of an infected person (2)
- ▢ Was kissed by an infected person (3)
- ▢ Sat or the lap of, or beside, an infected person (4)

Q53 When people in the household had COVID-19, approximately how much time did this cat spend in the room of an infected person on an average day?

- ◯ Less than 2 hours (1)
- ◯ 2-6 hours (2)
- ◯ 7-12 hours (3)
- ◯ 13-18 hours (4)
- ◯ 19-24 hours (5)

Q54 Around the time that people in the household had COVID-19, did this dog have any new occurrences of the following? (please check all that apply)

- ◯ Cough (1)
- ◯ Difficulty breathing (2)
- ◯ Vomiting (3)
- ◯ Diarrhea (4)
- ◯ Decreased appetite (5)
- ◯ Decreased energy (6)

End of Block: Block 4

Start of Block: Block 4
