# Supplementary material for: Risk Factors for SARS-CoV-2 Infection and Illness in Cats and Dogs
Source: Emerg Infect Dis. 2022 Jun;28(6):1154–62. doi: 10.3201/eid2806.220423 (PMC9155877; doi:10.3201/eid2806.220423)
Supplement: Appendix 3 — Questionnaire used in surveillance of SARS-CoV-2 infection, seropositivity, and illness in cats and dogs in an animal shelter. [file 22-0423-Techapp-s3.pdf]

Article DOI: https://doi.org/10.3201/eid2806.220423

Risk Factors for SARS-CoV-2 Infection and Illness in Cats and Dogs

Appendix 3

Questionnaire

The questionnaire given to owners of cats brought to a low-cost neuter clinic is reproduced on the following pages.

Toronto Animal Services COVID serology

Start of Block: Default Question Block

Q1 **Evaluation of antibodies against SARS-CoV-2, the cause of COVID-19, in cats** You are invited to participate in a study of SARS-CoV-2 infection in cats being coordinated by Drs. Scott Weese and Dorothee Bienzle of the Ontario Veterinary College, University of Guelph. The study aims to help us better understand transmission of this virus between people and their pets. Participants must be 18 years of age or older. This study is funded by the Public Health Agency of Canada.
If you agree to take part in this study, you will be asked to complete a short (5 minute) survey. If you wish to receive your cat's results, you will be asked to provide your name, phone number or email address. Otherwise, no identifying information will be collected. The questionnaire will ask about COVID-19 in your household and pet contact information. You will not be asked to identify specific people that are known or suspected to have COVID-19. This survey is voluntary and all identifying information will be treated as confidential. Only Drs. Weese and Bienzle will have access to the data. No identifying or individual results will be released and identifying information will be stored on an encrypted device. Although there is a risk that you may be concerned about how your responses will be evaluated or about providing information about COVID-19 diagnoses, there are no right or wrong answers. You will not be asked any details about anyone’s medical status beyond whether people in contact with the pet are known or suspected to be infected, and this information will be confidential. You will not be contacted about your survey results or participation, but will be informed of the testing results of your pet. By entering this survey, you indicate that you have read the information provided and consent to participate. There are no direct benefits to you, but results will improve our understanding SARS-CoV-2. You may choose to skip any question(s) you do not want to answer. You can stop completing the survey at any time. If you wish to have your answers removed or if you have any questions, you may contact Dr. Weese at 519-824-4120 ext 54064 or jsweese@uoguelph.ca. After July 1, 2021, we will not be able to remove data.
A summary of results will be available at http://www.wormsandgermsblog.com upon completion of the study and be part of a scientific paper. Data will be retained on a secure drive until publication of results, then survey results will be destroyed. We encourage you to print a copy of this consent page.
You do not waive any legal rights by agreeing to take part in this study. This study has been reviewed by the University of Guelph Research Ethics Board for compliance with federal guidelines for research involving human participants. If you have any questions about your rights as a research participant in this study (REB#20-04-002) please contact: Manager, Research Ethics, University of Guelph, reb@uoguelph.ca; 519-824-4120 ext 56606.By continuing on to the survey, you are indicating your consent to participate in this survey.

Q79 Would you like to receive results of your cat's test? If so, we will ask for your contact information.

○ Yes (1)

○ No (2)

Skip To: Q29 If Would you like to receive results of your cat's test? If so, we will ask for your contact informa... = No

Q2 What is your name?

____

Q3 What is the best phone number or email address to reach you at to provide you with test results?

____

Q29 Cat's name

____

Q83 Cat's age (or approximate age)

____

Q85 Cat's sex

○ Male (1)

○ Female (2)

Q84 Which best describes this cat?

- ◯ This is your cat (lives in the same household as you) (1)
- ◯ You are fostering this cat (3)
- ◯ This cat is currently at a shelter or rescue (4)
- ◯ Other (please specify) (5) ____

Q30 What best describes this cat

- ◯ Indoor exclusively (1)
- ◯ Mainly indoor with some outdoor access (2)
- ◯ Spends large amounts of time both indoors and outdoors (3)
- ◯ Mainly outdoor (4)
- ◯ Outdoor exclusively (5)
- ◯ Unknown background (6)
- ◯ Lives in a shelter (7)

Q5 How many people currently live in your household?

____

Q6 Was anyone in your household diagnosed with COVID-19 during the time the cat was present?

- Yes (4)
- No (5)
- Maybe, COVID was suspected but testing was not performed (6)
- Don't know/prefer not to answer (7)

Skip To: End of Block If Was anyone in your household diagnosed with COVID-19 during the time the cat was present? = No

Skip To: End of Block If Was anyone in your household diagnosed with COVID-19 during the time the cat was present? = Don't know/prefer not to answer

Q8 Approximately what date was COVID-19 first diagnosed or suspected in the household?

____

End of Block: Default Question Block

Start of Block: Block 4

Display This Question:

If Was anyone in your household diagnosed with COVID-19 during the time the cat was present? = Yes

Q34 When people in the household had COVID-19, approximately how much time did this cat spend in the room of an infected person on an average day?

- Less than 2 hours (1)
- 2-6 hours (2)
- 7-12 hours (3)
- 13-18 hours (4)
- 19-24 hours (5)

Display This Question:

If Was anyone in your household diagnosed with COVID-19 during the time the cat was present? = Yes

Q33 Around the time that people in the household had COVID-19, did this cat have any new occurrences of the following? (please check all that apply)

- ☐ Cough (1)
- ☐ Difficulty breathing (2)
- ☐ Vomiting (3)
- ☐ Diarrhea (4)
- ☐ Decreased appetite (5)
- ☐ Decreased energy (6)

Display This Question:

If Was anyone in your household diagnosed with COVID-19 during the time the cat was present? = Yes

Q45 When people in your household had COVID-19, which of the following likely occurred? (please check all that apply)

- ☐ Slept on/in the bed of an infected person (1)
- ☐ Licked the face or hands of an infected person (2)
- ☐ Was kissed by an infected person (3)
- ☐ Sat or the lap of, or beside, an infected person (4)

Q81 Thank you for your participation. Test results will be provided to you within a few weeks if you provided contact information. If you have any questions, please contact Dr. Scott Weese at jsweese@uoguelph.ca

End of Block: Block 4

Start of Block: Block 4
